# Design and Control of the Natural Frequency of Brake Discs in the Aspect of the Gray Cast Iron Production Process

**DOI:** 10.3390/ma17143490

**Published:** 2024-07-14

**Authors:** Andrzej Zyska, Mariusz Bieroński, Krzysztof Naplocha, Paweł Popielarski

**Affiliations:** 1 Faculty of Production Engineering and Materials Technology, Czestochowa University of Technology, 19 Armii Krajowej Av., 42-200 Czestochowa, Poland; 2 Brembo Poland Sp. z o.o., Roździeńskiego 13, 41-308 Dabrowa Gornicza, Poland; mariusz_bieronski@brembo.pl; 3 Department of Lightweight Elements Engineering, Foundry and Automation, Faculty of Mechanical Engineering, Wroclaw University of Science and Technology, 27 wybrzeże Stanisława Wyspiańskiego Str., 50-370 Wroclaw, Poland; krzyszof.naplocha@pwr.edu.pl; 4 Institute of Materials Technology, Poznan University of Technology, Piotrowo 3 Str., 61-138 Poznan, Poland; pawel.popielarski@put.poznan.pl

**Keywords:** brake discs, gray cast iron, mechanical properties, FRF analysis, numerical modal analysis

## Abstract

The results of research on the influence of the chemical composition of cast iron and its potential changes in the production cycle on the elastic properties and the correctness of numerical simulations of the natural frequency of ventilated brake discs are presented. The tests were carried out for three grades of gray cast iron with flake graphite with a eutectic saturation coefficient ranging from 0.88 to 1.01. A quantitative metallographic assessment of the pearlitic cast iron matrix and graphite precipitates was carried out, and the hardness and compressive/tensile strength of individual cast iron grades were determined, taking into account the limit contents of the alloying elements. Next, ultrasonic tests were performed, and the elastic properties of cast iron were determined. Based on the obtained data, a numerical modal analysis of brake discs was performed, the results of which were compared with the actual values of an FRF frequency analysis. The error of the computer simulations was estimated at approx. 1%, and it was found that the accuracy of the calculations of the first natural frequency did not depend on the dimensions (size) of the discs and the chemical composition of the cast iron from which they were cast. The functional relationships between the chemical composition of cast iron, its strength and elasticity and the first natural frequency of the disc vibrations were determined, and a database of the material parameters of the produced cast iron grades was developed. An implementation example showed the validation of the brake disc design with natural frequency prediction and demonstrated a high convergence of the experimental results with the simulated values. Using I-MR control cards, both the effectiveness of designing and predicting the natural vibrations of brake discs based on the implemented material database as well as the stability of the gray cast iron production and disc casting processes were confirmed.

## 1. Introduction

The basic element affecting the safety and comfort of driving a car is the disc braking system. The high power of the currently used drive units ensures a high driving speed, but at the same time requires meeting high and often contradictory requirements in terms of efficiency and braking quality. During car operation, brake discs are exposed to high and sometimes extreme thermal stresses. The temperature on the surface of the discs during sudden braking can reach values of 700 °C and sometimes even higher [1]. As a result, this leads to a change in the friction properties of the braking system and excessive wear. If the discs overheat during vehicle use and are unable to dissipate the accumulated friction heat, a breakdown occurs, i.e., brake fade [2]. Extreme thermal processes, however, can cause brake discs to deflect and crack. It is assumed that the wear of brake discs and the formation of a network of surface cracks are caused by low-cycle thermomechanical fatigue [3]. The processes of friction and wear of materials intended for brake discs have been the subject of numerous studies over decades [4,5,6]. As a result of this research, modern materials for brake discs have been developed, such as ceramic composites, metal composites reinforced with ceramic particles, carbon–carbon composites [6,7,8], as well as special coatings with high abrasion resistance [9,10,11]. Discs manufactured using modern composite materials and protective coatings are very expensive and can only be found in premium cars. In other car classes, the basic material for brake discs is still gray cast iron with flake graphite. The attractiveness and popularity of gray cast iron as a construction material result primarily from its low production cost and favorable set of physical and mechanical properties. Cast iron is characterized by high resistance to abrasion and thermal fatigue, including resistance to “thermal shocks”, as well as good thermal conductivity, hardness and tensile strength [12,13,14,15,16]. It also has very good castability, low solidification shrinkage, low tendency to hot cracking and good machinability and workability [16,17,18]. The main disadvantage of gray cast iron is its low corrosion resistance and relatively high density [16,19]. The microstructure of cast iron intended for casting brake discs consists of graphite precipitates and a pearlitic matrix. Pearlite provides the required level of mechanical properties [18,20,21], while graphite is a phase that significantly influences the friction processes in the brake pad–disc system [22]. Graphite reduces the coefficient of friction while increasing resistance to abrasive wear. During the operation of the brake and cyclically generated stresses, the graphite particles are crushed. In the friction node, the crushed particles are evenly distributed on the working track of the disc, having a protective effect on the metal matrix [22,23]. Graphite precipitates also play an essential role during extreme changes in brake disc temperature [24]. During rapid heating or cooling of the disc, the high coefficient of thermal expansion of the metal matrix contributes to the development of local thermal stresses. Graphite precipitates, due to their very low strength, are then freely deformed, compensating for the shrinkage and expansion effects [16]. Taking into account the comprehensive requirements regarding the mechanical properties, it was found that gray cast iron intended for casting brake discs should have a structure with graphite particles of medium size (100–200 μm) and uniformly distributed type A [25,26] (in accordance with the standard [27] 

In addition to the stringent safety requirements, the currently produced brake discs must have appropriate vibroacoustic features that determine the comfort of driving a car. In this respect, gray cast iron with flake graphite is also an irreplaceable material, because it has an exceptionally high vibration damping capacity among all technical metal alloys. The main problem that occurs during car operation, even in the initial period, are squeaks, noise and vibrations coming from the braking system. Although the issue of brake squealing has been researched for years, knowledge about the causes and mechanisms of its occurrence is quite limited. Research on disc brake assembly conducted by Liles [28] showed that brake squeaking increases with the increase in the coefficient of friction and the wear of friction materials, and in order to reduce it, shorter pads and a softer disc should be used. Research by Liu et al. [29] showed that brake noise and squeal can be reduced by increasing the stiffness of the disc, reducing the friction coefficient and using a damping material on the back side of the brake pads. In subsequent numerical and experimental works [30,31], it was found that the possibility of predicting and reducing unstable frequencies depends on the variability of the disc’s Young’s modulus.

The design of braking system structures with NVH (Noise, Vibration, Harshness) prediction is based on the finite element method. The most frequently used methods for predicting and eliminating noise are transient dynamic analysis and complex eigenvalue analysis [29,31,32,33]. Based on numerical simulations, the frequency characteristics of the disc brake assembly are optimized, and the natural vibration range of its individual elements is determined. The general concept of designing the acoustic properties of the braking system as well as of the entire car is to bring various sound sources into a harmonious, well-balanced overall sound. Optimizing individual sources in itself does not automatically result in an ideal overall vehicle sound [34].

During the production of brake discs, in order to ensure their high quality and meet the acceptance criteria regarding natural frequencies, a number of control tests are carried out, including FRF analysis (Frequency Response Function). Experimental research on natural vibrations is of particular importance at the stage of implementation of new brake discs and discs already produced for which a structural modification is planned. Then, FRF analysis allows for the validation of the numerical modal model of the shield and the optimization of its geometry. Determining the permissible minimum and maximum NVH frequencies of a disc cast from a specific type of gray cast iron is related to the performance of many design and technological tasks at the foundry, including the preparation of a 3D design of a raw casting of a prototype of the disc, the concept of the casting method and the execution of molding and foundry molds, carrying out preliminary melts with appropriate chemical composition. Then, the cast discs are mechanically processed, and the actual natural frequency is determined using the FRF method. The results of experimental modal analysis usually differ from the simulation values, which consequently results in the need to make adjustments to the disc structure and requires the re-implementation of the design and technological tasks necessary for experimental validation.

The aim of this research was to assess the impact of the chemical composition of cast iron and its potential changes during the production cycle on the elastic properties and correctness of the numerical solution of the natural vibrations of ventilated brake discs. In this work, we assumed that the adequacy of the results of the disc modal model depends on the method and the accuracy of the measurement of the elastic parameters of cast iron used in the calculations. Determining the appropriate NVH frequency range by means of a numerical design therefore requires the development of precise material databases for the produced cast iron grades. Based on the created database, it will be possible to determine the relationships between the chemical composition of cast iron, its strength and elasticity and the natural frequency of the discs. Using the determined values of the material parameters, validation of the disc geometry can be carried out using numerical modal analysis, as a result of which the final shape of the brake disc can be selected. The tests were planned for three types of cast iron, from which the considered foundry produces approximately 70% of all brake discs. In order to ensure high accuracy of the results and to link the elastic parameters with the cast iron melting process and disc casting, the elastic modulus and Poisson number were measured directly on the brake discs using the ultrasonic method.

## 2. Research Material and Research Methodology

Computer simulations and experimental measurements were performed in industrial conditions, in a foundry producing braking system components for passenger cars. Two ventilated brake discs with the largest and smallest diameters produced by foundries were selected for testing. The 3D model of the cast discs and their characteristic dimensions are presented in Table 1.

The tests were carried out for three grades of cast iron with a tensile strength in the range of 150–250 MPa. Three melts were made for each grade of cast iron, producing cast iron with an optimal composition and compositions with the permitted minimum and maximum content of the alloying elements, the limit values of which are specified by company standards. The chemical composition of the individual cast iron grades and their degree of eutectic saturation are presented in Table 2.

**Figure 1:**
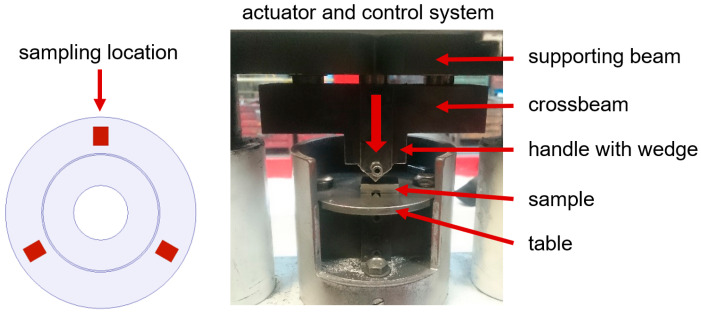
Station for measuring strength using the “wedge method” and place where the samples were positioned.

Cast iron was melted in a crucible induction furnace with an inert lining, with a power of 8 MW and a capacity of 17 Mg. While pouring the molds, the cast iron was modified using the “liquid metal jet” method and the Superseed modifier with a particle size of 0.2–0.7 mm. The tests were carried out on 180 brake discs, casting 10 discs no. 1 and 10 discs no. 2 from each melt. The following measurements were made directly on the cast brake discs: compressive/tensile strength, hardness and propagation speed of longitudinal and transverse waves. Based on the results of the ultrasonic tests, the values of Young’s modulus and Poisson’s number were calculated. Then, based on the obtained material data, numerical simulations of the brake discs’ natural vibrations were performed. Strength tests were performed using the “wedge method” on the measuring station shown in Figure 1.

Test samples were taken from three areas of the disc raceway (Figure 1) and mechanically processed into cuboids with dimensions of 6 mm × 20 mm × 25 mm. The processed samples were placed in the working space of the machine between the wedge and the table and then subjected to uniaxial compression until they broke. During the test, the compressive strength *R_mK_* was recorded and determined, and then the obtained results were converted into tensile strength *R_m_* using the relationship:

(1) $$R_{m}=1.8\times R_{mK}\;–55\;(MPa)$$

Equation (1) is a regression equation (empirical) and was developed on the basis of the foundry’s own research by correlating the measurement results from a standard tensile test with the results of compressive strength tests using the “wedge method”. For cast iron grades with a tensile strength ranging from 150 to 300 MPa, the conversion error of the measurement results does not exceed 5 MPa. The adopted methodology of strength testing is related to the conditions for accepting brake discs imposed by the foundry’s contractors.

The hardness measurements of the castings were carried out using the Brinell method in accordance with the standard [35]. The tests were carried out using a sintered carbide ball with a diameter of 5 mm, with four measurements performed on the disc raceways for each casting.

The microstructural tests of the brake discs were carried out based on the ISO 945 standard on a light microscope with Olympus Stream Essentials software (Version 2.2). The research assessed the size and form of the graphite precipitates and metal matrix, using magnifications of 100× and 500×, respectively.

**Figure 2:**
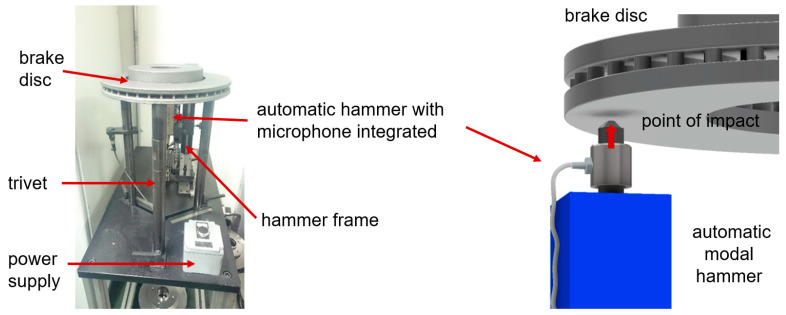
Itasonic station and natural frequency measurement diagram.

**Figure 3:**
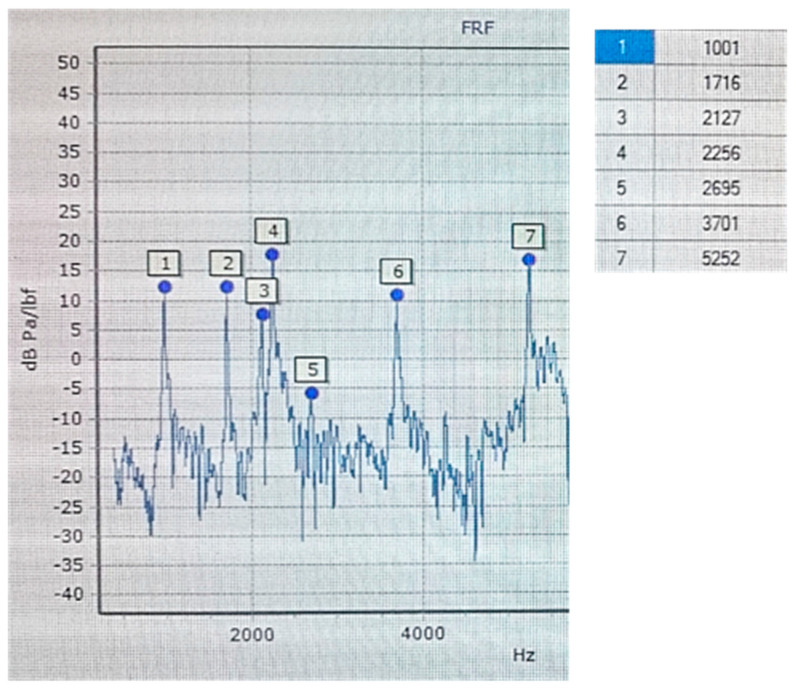
Measured frequency response function (FRF), cast iron grade C, type OPT, disc No. 1.

An Olympus (Shinjuku, Japan) DL 38 Plus device equipped with two broadband heads, VL-M110-RM and VT-V156-RM, with a nominal characteristic of 4 MHz was used for the ultrasonic tests. The VL-M110-RM heads were used to measure the longitudinal wave speed, while the VT-V156-RM heads was used to measure the transverse wave speed. The measuring device and heads used complied with the standards [36,37]. In order to determine the elastic properties of cast iron, density measurements were also carried out. The tests were carried out using the hydrostatic weighing method according to the standard [38]. Samples with dimensions of 5 mm × 10 mm × 10 mm were taken randomly from castings of each grade of cast iron and prepared on a CNC machine. The numerical modal analysis of the brake discs was carried out in the MSC Nastran simulation package. Actual tests of the natural frequency of brake discs (FRF analysis) were performed at the Itasonic 2010 station. The experimental modal analysis used involved forcing natural vibrations using a modal hammer and then recording and identifying the frequency response spectrum. The casting was vibrated with an automatic modal hammer of 0.1 kg and a head diameter of 15 mm. The emitted sound signal was recorded using a special industrial microphone with a frequency response of 100 Hz–10 kHz. The method for measuring the natural vibrations and an example graph from the frequency analysis (FRF) are shown in Figure 2 and Figure 3.

## 3. Research Results and Discussion

### 3.1. Mechanical Properties Testing

**Figure 4:**
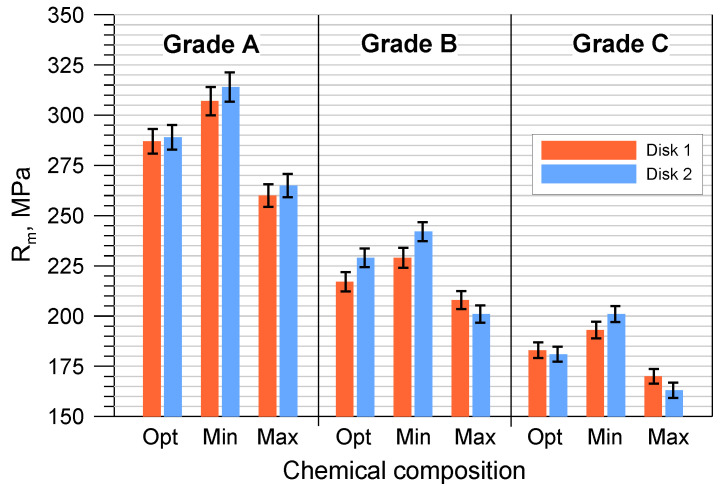
Tensile strength of the tested gray cast iron grades.

**Figure 5:**
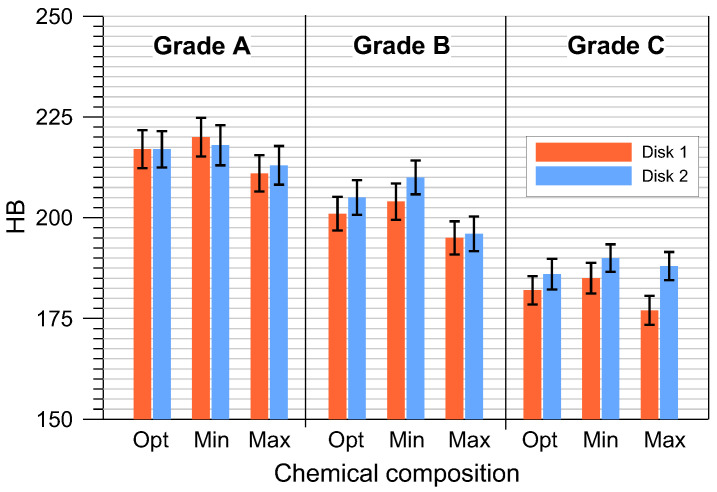
Hardness of the tested gray cast iron grades.

The average hardness and tensile strength of the tested cast iron grades with optimal compositions and the permissible minimum and maximum element contents are shown in Figure 4 and Figure 5. The tensile strength was determined indirectly on the basis of the *R_mK_* test results according to the methodology described in point. Based on the tests performed, it can be concluded that the highest hardness and tensile strength were obtained for grade A cast iron, and the lowest for grade C. At the same time, it should be emphasized that all manufactured grades met the requirements for minimum tensile strength (Grade A > 250 MPa, Grade B > 200 MPa and Grade C > 150 MPa). Analyzing the results in more detail (Figure 4 and Figure 5), it can be seen that the highest HB and *R_m_* values corresponded to cast iron grades with the chemical composition MIN. Increasing the permissible element content to the MAX value resulted in a noticeable reduction in mechanical properties. In the case of grade A cast iron, the tensile strength decreased by approximately 45 MPa, and the hardness decreased by 7 HB. For B and C grade cast iron with MAX chemical composition, these properties decreased by 31 MPa and 14 HB and by 34 MPa and 11 HB, respectively.

The determined changes in the mechanical parameters and their distribution were directly related to the degree of eutectic saturation S_C_ of the individual cast iron grades and are important both for the design of brake discs and for the melting of cast iron. As is known, as the S_C_ coefficient increases, the tensile strength and elastic properties of cast iron decrease, while its ability to dampen vibrations and resistance to thermal fatigue increases [16,18,27]. A comprehensive analysis of these properties, taking into account the technological aspects of cast iron melting, allows for the determination of critical values for which the designed brake discs meet the desired braking performance and appropriate NVH properties. Smelting cast iron for brake discs requires a high technological regime and constant control in order to maintain a narrow range of change for the S_C_ coefficient—on average of ±0.02 (Table 1). Therefore, for the smelting of cast iron, a high-quality metal input is used, containing hematite pig iron, selected steel and circulating scrap, and certified and proven auxiliary agents (ferroalloys and carburizers). Moreover, in order to ensure a uniform structure and mechanical properties of the casting, as well as to reduce the tendency to whitening and the segregation of components, cast iron is modified when poured into molds.

### 3.2. Results of the Microstructural Tests

**Figure 6:**
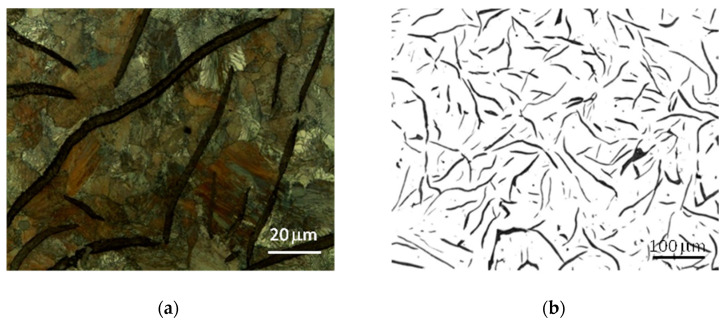
Microstructure of cast iron grade A: (**a**) sample etched with nital 4%, (**b**) sample without etching.

**Figure 7:**
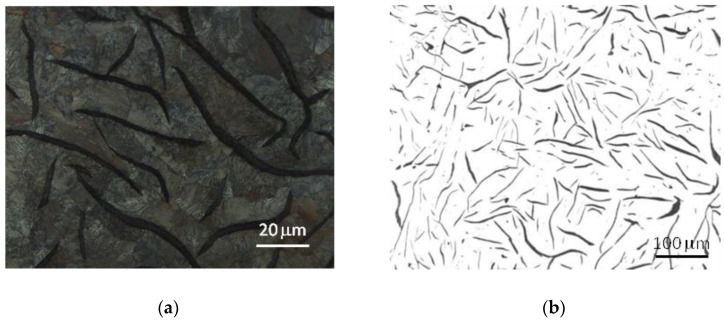
Microstructure of cast iron grade B: (**a**) sample etched with nital 4%, (**b**) sample without etching.

**Figure 8:**
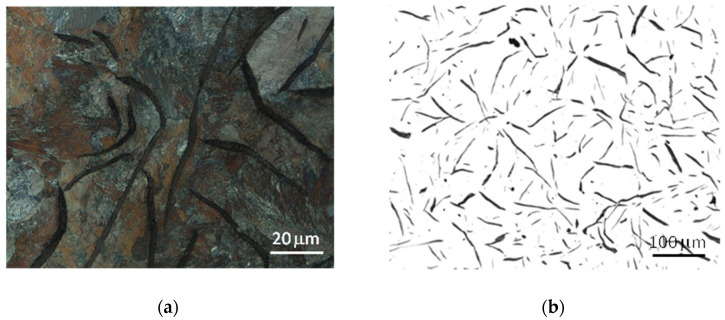
Microstructure of cast iron grade C: (**a**) sample etched with nital 4%, (**b**) sample without etching.

The microstructure of the tested cast iron grades and the measurements of the morphological characteristics of the graphite precipitates and matrix material are presented in Table 3 and Figure 6, Figure 7 and Figure 8. The analyzed grades were characterized by quite similar chemical compositions; therefore their microstructures were very similar. All grades of cast iron were dominated by simple, medium-sized flake graphite, whose distribution in the metal matrix was uniform. No non-metallic inclusions, impurities or microporosity were found in the structure of the material. By performing a more detailed assessment of graphite based on quantitative metallographic tests, it could be concluded that in terms of distribution, size and shape, the observed precipitates showed the following features:

- The graphite distribution was uniform—type A (70–75%) and interdendritic types D and E (19–26%);
- The size of the graphite precipitates corresponded to the size patterns from number 3 to number 5. The largest share, over 90%, consisted of graphite precipitates corresponding to the length patterns 4 and 5 (from 60 to 250 μm);
- The graphite precipitates had a straight flake shape and corresponded to pattern I.

The tested cast iron grades had a pearlite matrix with a medium degree of dispersion and, using the marking according to the standard [39], the amount of pearlite in all grades was P96.

Despite the very high microstructural similarities of the studied cast iron grades, the metallographic measurements revealed a tendency for graphite precipitation to decrease with the increase in the degree of eutectic saturation. In grade A cast iron, the share of graphite with a size of 60–120 μm (pattern 5) was approximately 60%, while in grade C cast iron, this share was 10% higher. It should also be noted that the microstructural features of the cast iron components corresponded to the company standards established jointly with the foundry’s contractors and were also consistent with the general requirements included, among others, in [40].

### 3.3. Testing the Elastic Properties of Cast Iron Using the Ultrasonic Method

The tests of elastic properties were preceded by density measurements (Table 4), which were performed using the hydrostatic weighing method. All samples were weighed in air and water, and then their density was determined based on the relationship:

(2) $$\rho_{P}=\frac{m_{1}}{m_{1}-m_{2}}\times \rho_{W}$$

where *ρ_P_*—sample density; *m*_1_—sample weight in air; *m*_2_—weight of the sample in water; and *ρ_W_*—density of water.

The ultrasonic method was used to determine the elastic modulus *E* and Poisson’s number *υ*. The average values of the velocities of longitudinal waves and transverse waves measured for the individual cast iron grades are presented in Table 5. Based on the obtained results of the ultrasonic tests and the density measurements, the parameters *E* and *υ* were calculated using Equations (3) and (4) [41]:

(3) $$v=\frac{1-2\;{\left(V_{L}/V_{S}\right)}^{2}}{2-{2(V_{L}/V_{S})}^{2}}$$

(4) $$E=\frac{{{\rho V}_{L}}^{2}(1+v)(1-2v)}{\left(1-v\right)}$$

where *V_L_*, $V_{S}$—velocities of longitudinal and transverse waves, respectively, and *ρ*—density.

The ultrasonic measurements of the brake discs and the calculation results obtained based on Equations (3) and (4) showed that for the tested cast iron grades with a eutectic saturation level from 0.88 to 1.01, the elastic modulus varied in the range of approx. 100–140 MPa. The highest E modulus value was found for grade A cast iron, which was also characterized by the highest strength, while the lowest Young’s modulus values were found for grade C cast iron, with a high S_C_ coefficient and the lowest mechanical properties.

A characteristic feature of the research methodology used is the small standard deviation of the E modulus, which was 2.3–4.3 GPa. A high accuracy of the results as well as the possibility of direct measurements on castings are an important advantage of the ultrasonic method. This allows for the adoption of reliable and real material data for numerical frequency analyses, as well as for the control of the brake disc production process. It should be noted that the elastic modulus is a material property and mainly depends on the number, shape and size of the graphite precipitates and the type of the cast iron matrix [16,18]. The second value assessed in the ultrasonic tests, i.e., the Poisson number, was very similar for all types of cast iron and was in the range of 0.26–0.27.

### 3.4. Simulations and Experimental Tests of the Natural Frequency of Brake Discs

A numerical modal analysis of the target castings was performed in the MSC Nastran simulation program. The MSC Nastran software (Version 2017) has an integrated set of tools for designing and optimizing the structure of braking systems based on a comprehensive simulation of the braking dynamics. As a result of the calculations, the vibroacoustic characteristics of the entire braking system and its individual elements are determined. In relation to brake discs, modal simulations allow for the verification of the disc geometry and the determination of the correct NVH vibration range. The calculations as well as the experimental tests focused on the assessment of the first (lowest) natural frequency of vibrations as the basic parameter for the reception of brake discs by foundry contractors. This parameter is strictly controlled in the production process (FRF analysis), and exceeding its limit values results in the disc being rejected for re-melting. Modal simulations were carried out based on the implemented material parameters *E*, *υ* and *ρ*, which were determined in the ultrasonic and hydrostatic tests. In the MSC Nastran solver, these data were used to build mass and stiffness matrices. Three-dimensional models of the cast discs were made in the CAD module, and then a finite element mesh was generated consisting of approx. 350,000 elements for disc No. 1 and approx. 400,000 elements for disc No. 2. Modal simulations were carried out assuming a non-contact boundary condition, which is adequate to the methodology of testing natural vibrations using the Itasonic apparatus.

**Figure 9:**
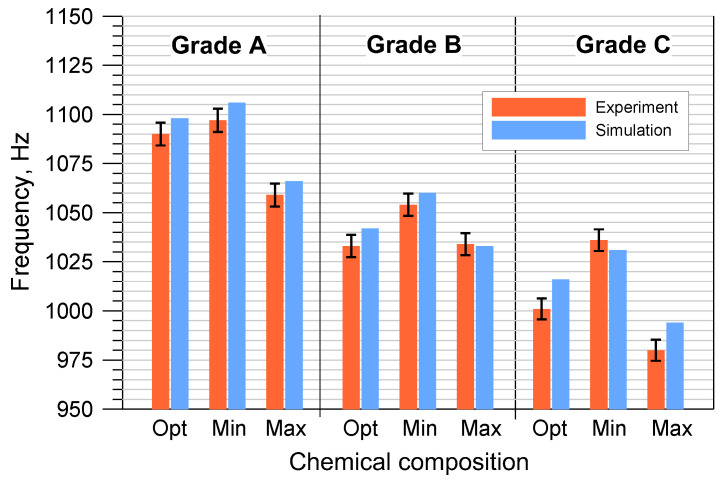
The first natural frequency of disc No. 1 cast from 3 grades of cast iron with the Opt/Min/Max composition.

**Figure 10:**
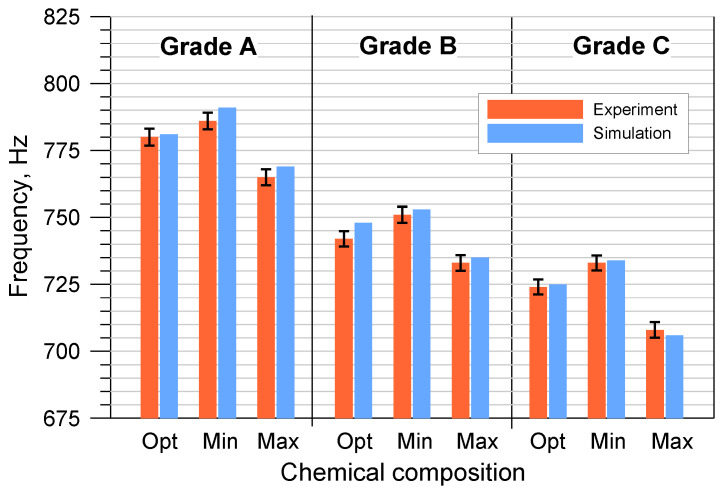
The first natural frequency of disk No. 2 cast from 3 grades of cast iron with the Opt/Min/Max composition.

The results of the numerical modal analysis and the experimental measurements of the discs cast from the tested cast iron grades, taking into account the permissible minimum and maximum element contents, are presented in Figure 9 and Figure 10.

The performed tests clearly revealed a strong relationship between the first natural frequency of the brake discs and the degree of eutectic saturation. As the carbon content in cast iron increased, its ability to dampen vibrations increased, which directly affected the vibration frequency of the brake discs. Castings made of cast iron grades with a higher S_C_ coefficient are always characterized by a lower natural frequency. When assessing all grades of cast iron with OPT composition, the first natural frequency of disc No. 1 ranged from 1001 to 1090 Hz, while that of disc No. 2 ranged from 724 to 780 Hz.

From the point of view of the production of brake discs and cast iron smelting, changes in chemical composition within the MIN-MAX limit range are important. The obtained results indicate that the differences in natural frequency caused by the deviation of the chemical composition from the optimal one ranged from several to several dozen hertz. The sensitivity of the natural frequency to changes in the S_C_ coefficient requires strict control in the selection of the metal charge as well as in the melting of cast iron. The permissible range of changes in the first natural frequency is agreed individually with the foundry’s contractors, but most often it is 3–5% compared to the value specified in the order. It should be emphasized that in the case of the tested disc castings, all results fell within the required frequency ranges.

Analyzing Figure 9 and Figure 10, it can also be concluded that the numerical simulations of the natural frequency of the brake discs showed a high convergence with the experimental results. The own material parameters used for the calculations ensured a high reliability of the simulation. The differences between the empirical values of the first natural frequency and the values obtained from the modal analysis were most often of several hertz. In order to more precisely assess the correctness of the numerical solution, the percentage errors of individual simulation results were determined:

(5) $$\Delta_{S}=\left|\frac{I_{S}-I_{R}}{I_{R}}\right|\times 100\%$$

where *I_S_*—value from the numerical simulation, and *I_R_*—value from the experiment

The calculations presented in Table 5 show that the size of the simulation errors, apart from two cases, did not exceed 1%. The average value of Δ*_S_* was 0.63%, and the maximum error reached 1.5%. The high convergence of the simulation results with the experimental measurement results also indicates the high accuracy and usefulness of the ultrasonic method for determining the elastic modulus and Poisson number. This is particularly important in relation to cast iron grades that differ quite slightly in the eutectic saturation coefficient S_C_.

### 3.5. Development of a Production Material Database

**Figure 11:**
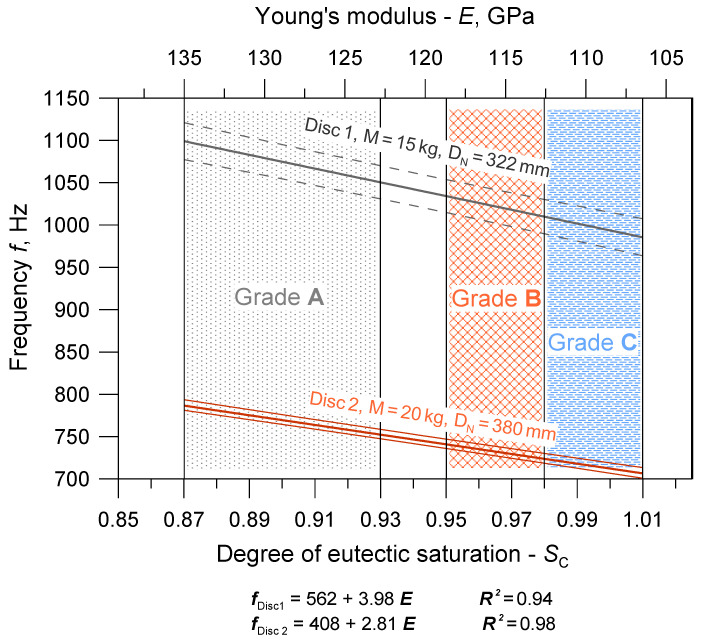
Relations between the chemical composition of cast iron, Young’s modulus and the first natural frequency for ventilated brake discs.

**Figure 12:**
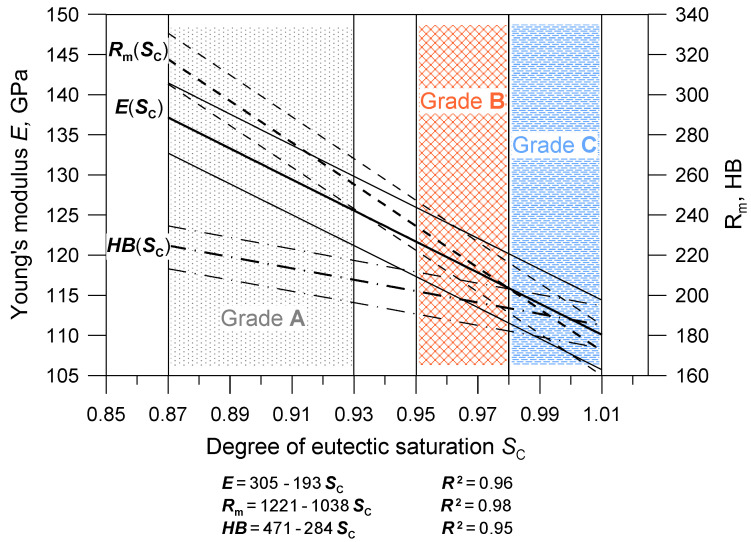
Functional dependencies between the chemical composition of cast iron and its mechanical properties.

Based on the obtained research results, a database of cast iron material parameters was developed, intended for the numerical simulation of the natural frequency of brake discs in the Nastran software. The database includes average values of parameters and their ranges of changes corresponding to the limit chemical compositions of individual cast iron grades. An integral part of the database are the two charts shown in Figure 11 and Figure 12. They illustrate the relationships between the chemical composition, strength and elasticity of cast iron and the natural frequency of the discs. The areas highlighted in the drawings (hatched fields) determine the possible ranges of changes in the material parameters for discs cast from particular grades of cast iron. At the stage of developing the production database, functional relationships were determined between first natural frequency, chemical composition and mechanical properties of the tested cast iron grades. Correlations related to Poisson number were not determined because the υ values varied within too narrow a range. When starting the statistical analysis, it was assumed that from among various functions approximating the relationships *f*(E), *E*(S_C_), *R*_m_(S_C_), *HB*(S_C_), the simplest formulas would be selected, for which the coefficient of determination R^2^ would be above 95%. The form of the obtained functions along with the values of the R^2^ coefficients and confidence interval (0.95) are presented in Figure 11 and Figure 12.

The presented charts are important from a practical point of view. On their basis, it is possible to initially predict the first natural frequency of ventilated brake discs depending on the chemical composition of cast iron and its strength. You can also make the opposite forecast, i.e., select the chemical composition of cast iron for a given type of disc and its natural frequency. Figure 11 and Figure 12 also show that a larger mass and nominal diameter of the disc correspond to lower values of the first natural frequency, which is a general guideline when predicting the NVH frequency and designing the disc geometry. It should be emphasized, however, that the basic quantity used for the numerical modal analysis and, consequently, for the optimization of the disc geometry was Young’s modulus.

### 3.6. Predicting the Natural Frequency when Implementing a New Brake Disc

#### 3.6.1. Design and Simulation Work

**Figure 13:**
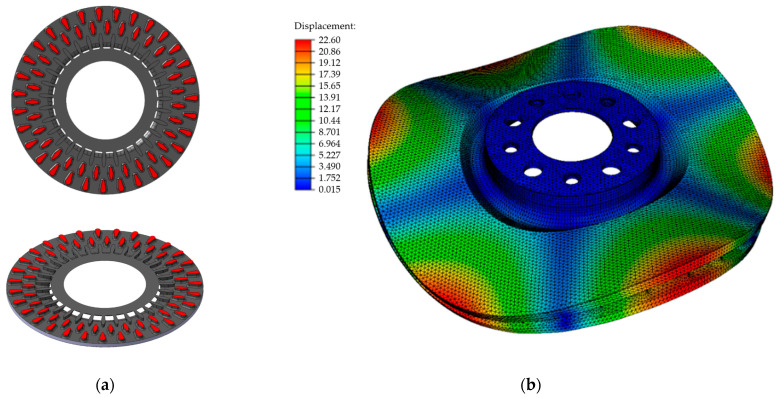
Disc before modification: (**a**) shape and arrangement of the blades in the space between the discs, (**b**) form of the natural vibrations for the second natural frequency of 1676 Hz.

The effectiveness of predicting the natural frequency of brake discs based on our own database of material parameters was verified in production conditions by fulfilling an order for a pilot series of disc castings placed by a new foundry customer. The order concerned the production of ventilated brake discs with a nominal diameter of 358 mm and a weight of 15.2 kg. The technical documentation included a construction drawing of the disc casting, the permissible range of the first natural frequency (770 ± 30 Hz) and the minimum level of tensile strength, i.e., −200 MPa. The characteristic dimensions of the disc were also specified, such as disc thickness, total disc thickness, nominal diameter, etc., which were final and could not undergo any changes. Any corrections could be made to the geometry and arrangement of the blades connecting the discs of the brake disk. The construction of the ordered disc included, among others oblique and oval blades arranged in two rows in a circular formation. The shape and arrangement of the blades in the interdiscal space are shown in Figure 13.

The prediction of the natural frequency began with selecting the cast iron grade and analyzing the disc structure. In order to meet the strength requirements as well as other acceptance criteria (specified by the client), grade B cast iron was selected for the disc castings. Next, a pre-processing procedure was carried out in the Nastran software, the solid model of the disc provided by the client was checked, and the material parameters of the cast iron from the production database and modal analysis were used in the program’s simulation module. Average values for the optimal chemical composition of cast iron were adopted for the calculations. The results of the computer simulations of the first natural frequency of the disc as well as of the frequencies of higher modes are presented in Table 6. In production conditions, basic acoustic tests focus on the analysis of the first natural frequency (due to the acceptance criterion of the discs), but at the stage of computer simulations it is also possible to determine and compare higher order modes for various design solutions. The form of the disc’s natural vibrations for the second natural frequency is shown in Figure 13b.

**Figure 14:**
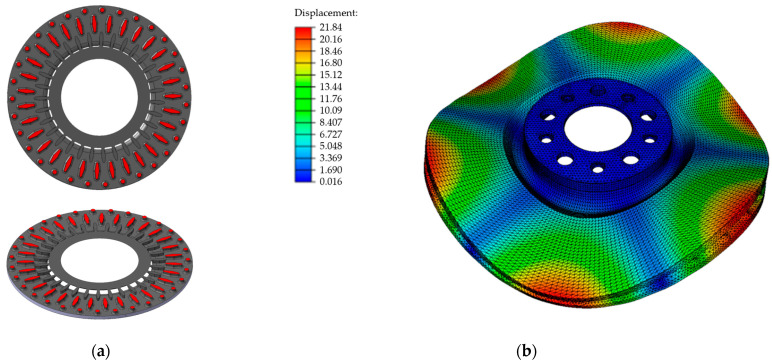
The disc after modification: (**a**) shape and arrangement of the blades in the space between the discs, (**b**) form of natural vibrations for the second natural frequency of 1851 Hz.

The calculations (Table 6) show that the first natural frequency of the disc designed by the client was significantly beyond the permissible reception range (770 ± 30 Hz). In order to obtain the desired NVH frequency values, the blades were corrected, introducing greater differences in their shape. For the outer array, circular sections were used, while in the central array, the oval cross section was retained, but its size was increased. The final dimensions of the individual blades were determined by simulating the brake disc’s natural vibrations several times. The shape of the designed blades and their arrangement in the interdiscal space are shown in Figure 14a. The results of the numerical modal analysis for the developed disc structure are presented in Table 6 and Figure 14b. Table 6 also shows the differences between the natural frequency of the disc with the original geometry and that of the modified disc, taking into account first- and higher order modes. The simulations carried out allowed us to design the final structure of the brake disc, for which the first natural frequency was in the middle of the frequency range 740–800 H.

#### 3.6.2. Experimental Verification of the Modal Simulation Results

In order to verify the calculation results, experimental tests were performed using the methodology described in the previous sections. A model plate was designed and manufactured, cast iron with OPT (optimal) composition was melted, and then a series of mechanical tests and FRF frequency analyses were carried out. The measurement results, together with the estimated error of computer simulations and the error of calculation results obtained from the regression function, are presented in Table 7. The error of the regression function was determined in a manner analogous to that used for the error of the computer simulations (Equation (5)).

The error values Δ*_S_* showed high compliance between the results of the theoretical and the experimental studies. The average simulation error of the first natural frequency was 0.65% and approximately corresponded to the value determined in the basic research. The predicted values of tensile strength, hardness and Young’s modulus also corresponded to the actual measurements.

### 3.7. Production Control of the Implemented Brake Disc

**Figure 15:**
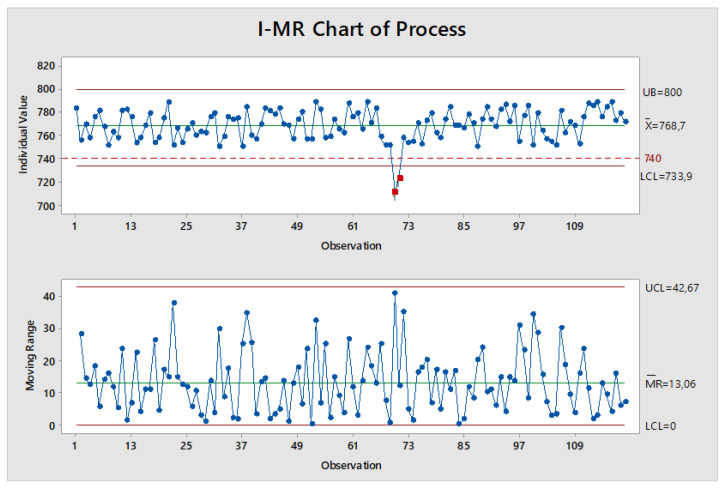
I-MR control card for the first natural frequency of the newly implemented brake disc, (dashed line-lower limits of the NVH frequency, red points-measurements outside the permissible range).

In order to more precisely verify the calculation results presented in the previous point, as well as to assess the stability of the production process, statistical quality control was performed using the I-MR (Individual–Moving Range) card. The monitored parameter was the first natural frequency of the implemented disc, the variability of which was determined on a sample of 120 castings. The measurements were carried out on castings selected randomly from the monthly production period. The developed I-MR control card is shown in Figure 15. The drawing also includes the upper and the lower limits of the NVH frequency range specified by the foundry’s customer.

The obtained sample characteristics (Figure 15) did not reveal, apart from those in the middle period, any disruptions in the analyzed production period. Using the guidelines specified in the standard [42], there were no sequences of points that signaled process irregularities. Although, according to the I-MR card, the brake disc production process could be considered stable, two measurements were recorded that exceeded the permissible NVH frequency range. A closer analysis of the NVH frequency failure showed that the measurements were performed on discs cast during the production period when there were problems with dissolving the pellet carburizer in the Junker furnaces.

## 4. Summary and Concluding Statements

The main goal of the research was to assess the impact of the chemical composition of cast iron and its potential changes during the production cycle on the elastic properties and the correctness of the numerical solution of natural vibrations of ventilated brake discs. A measurable economic effect of the research is the elimination, at the implementation stage, of a number of design and technological works related to the production of brake disc prototypes and the experimental determination of their geometry that meets the desired natural vibration frequency (NVH) range. The experiments were carried out for three grades of cast iron with the degree of eutectic saturation varying from 0.88 to 1.01 and tensile strength from 150 MPa to 250 MPa. These types of cast iron grades are most often used by automotive manufacturers for brake disc castings. In order to ensure a high precision of the results and to take into account the influence of technological factors on the properties of cast iron, it was assumed that all measurements would be made directly on the castings. The strength tests performed using the wedge method and the hardness tests showed that these properties depend on the chemical composition of cast iron; the influence of the mass, size and geometry of the disc was insignificant in the analyzed range of changes. The cast iron melting process together with the modification procedure ensured the obtaining of the correct microstructure, which was very similar for all grades and was characterized by a pearlitic matrix (class P96) and simple, medium-sized flake graphite with uniform distribution (70–75% type A). One of the important features determined and assessed as part of our research was the elastic modulus.

The accuracy of the E modulus measurement determines the adequacy of the modal model of the brake disc. The obtained results showed that the ultrasonic method selected for testing was very effective in production conditions. It allowed for accurate and quick measurements of the propagation speed of longitudinal and transverse ultrasonic waves directly on brake disc castings, and the values of elastic modulus and Poisson number calculated on their basis ensured a high convergence of the results of the natural vibration simulations with the results of the FRF frequency analysis. The average error of the modal simulations was 0.63%, while the maximum error was 1.5%.

In the next stage of the research, regression analysis was carried out, and linear relationships were determined between Young’s modulus and the first natural frequency of vibrations of solid and ventilated brake discs, and between Young’s modulus and the tensile strength, hardness and degree of saturation of eutectic cast iron. For the tested cast iron grades, the Young’s modulus value varied in the range from approximately 100 to 140 GPa, with the lowest values for cast iron with a eutectic saturation degree of 1.01. As the E modulus increases, the tensile strength of cast iron, its hardness and the first natural frequency of brake discs increase, regardless of the disc shape and dimensions. The intensity of these interactions is described by the appropriate values of the directional coefficients of the linear equations presented in Figure 11 and Figure 12.

The obtained test results and statistical analysis results made it possible to develop a database of the material parameters of the analyzed cast iron grades. This database was implemented into the MSC Nastran software environment and used to simulate the natural vibrations of the newly implemented brake disc. As a result of the modal simulations, the original geometry of the disc was verified, and the final shape and arrangement of the blades in the interdiscal space were designed.

The correctness of the design solution as well as the reliability of the numerical simulations confirmed the results of the experimental tests. The obtained *δ_S_* error value for the described implementation was 0.65%. High consistency of the results was also obtained in terms of predicted material parameters. The errors in the calculations of tensile strength, Young’s modulus and hardness were in the range of 0.4–2%. Subsequent tests performed using the I-MR control card showed high stability of the production process of the implemented brake disc. The obtained course of I-MR variability proved that the design and simulation work, as well as the technological work (melting cast iron with a strictly controlled chemical composition) were carried out correctly and that the developed production material database can ensure the effective prediction of the natural vibration frequency of newly designed brake discs without the need for casting modal prototypes.

### Final Statements

1. An increase in the degree of saturation of eutectic cast iron from 0.87 to 1.01 resulted in a 21% reduction in Young’s modulus and a 10–11% reduction in the first natural vibration frequency of the brake discs.
2. During the production cycle, even slight changes in the chemical composition of cast iron may affect the frequency stability of the discs. The high sensitivity of the first natural frequency to changes in the S_C_ coefficient requires the use of high-quality input materials and strict process control at the stages of melting and modification of cast iron.
3. The numerical prediction of the first natural frequency and the range of its changes are determined by the accuracy of the measurement of the elastic properties of cast iron. The ultrasonic method based on measuring the propagation speed of longitudinal and transverse waves allows for a quick and accurate determination of Young’s modulus and Poisson number of brake discs.
4. For the analyzed brake disc geometries and cast iron grades (S_C_ from 0.88 to 1.01), the relationships between the first natural frequency and Young’s modulus describe linear functions with direction coefficients ranging from 2.8 to 4.0 Hz/GPa
5. Carrying out design and simulation work using the production material database is an effective method for optimizing the design of brake discs. The error of the numerical simulations of the first natural vibration frequency was on average below 1%.
6. Control tests performed using I-MR cards confirmed both the effectiveness of the design and prediction of natural vibrations of brake discs, as well as the stability of the gray cast iron production and disc casting processes.

## Figures and Tables

**Table 1:** Dimensions of the analyzed castings.

	Disc Code	Outer Diameter [mm]	Inner Diameter [mm]	Thickness [mm]	Mass [kg]
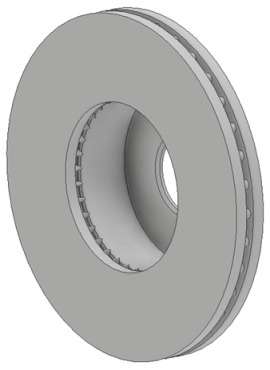	Disc 1	322	191	32	15
	Disc 2	380	221	34	19.9

**Table 2:** Chemical composition of three grades of cast iron used for brake discs.

Cast Iron Grade	Type of Chemical Composition	Chemical Composition, wt.%										Degree of Eutectic Saturation S_C_	Carbon Equivalent (CE)
		C	Si	Mn	P	S	Cu	Cr	Mo	Ni	Sn		
A	MIN	3.25	1.70	0.50	0.00	0.05	0.10	0.13	0.00	0.00	0.00	0.87	3.78
	OPT	3.35	1.75	0.60	-	0.08	0.15	0.15	-	-	-	0.90	3.90
	MAX	3.40	1.80	0.70	0.10	0.10	0.25	0.20	0.20	0.20	0.09	0.93	3.99
B	MIN	3.67	1.40	0.55	0.00	0.00	0.30	0.25	0.00	0.00	0.00	0.95	4.07
	OPT	3.70	1.45	0.60	-	0.08	0.32	0.25	-	-	-	0.97	4.12
	MAX	3.72	1.50	0.70	0.10	0.15	0.35	0.35	0.15	0.10	0.09	0.98	4.16
C	MIN	3.70	1.60	0.50	0.00	0.00	0.15	0.13	0.00	0.00	0.00	0.98	4.18
	OPT	3.73	1.65	0.55	-	0.08	0.20	0.15	-	-	-	1.00	4.25
	MAX	3.75	1.70	0.75	0.08	0.10	0.25	0.25	0.10	0.10	0.09	1.01	4.29

**Table 3:** Quantitative tests of graphite precipitates and pearlitic matrix.

Cast Iron Grade	Disc Code	Graphite							Metal Matrix		
		Shape	Distribution, %			Length, %			Perlite, %	Ferrite, %	Cementite, %
			A	B	D–E	5	4	3			
A	Disc 1	I	71	4	25	59	36	5	93	6	<1
	Disc 2	I	75	4	21	63	31	6	95	4	<1
B	Disc 1	I	72	4	24	63	32	5	94	5	<1
	Disc 2	I	75	4	21	65	29	6	95	4	<1
C	Disc 1	I	72	4	24	70	24	6	93	6	<1
	Disc 2	I	76	3	21	74	22	4	95	4	<1

**Table 4:** Results of the ultrasonic tests and determined elastic parameters of the tested cast iron grades.

Cast Iron Grade	Chemical Composition	Disc Code	Average Speed (mm/µs)		E (GPa)	E According to Chemical Composition (GPa)	Standard Deviation σ(E)	Poisson’s Number	Density, g/cm^3^
			V_L_	V_S_					
A	MIN	Disc 1	4.87	2.71	135	135	3.15	0.26	7.192
		Disc 2	4.84	2.72	135				
	OPT	Disc 1	4.77	2.67	131	131.5		0.26	7.188
		Disc 2	4.78	2.69	132				
	MAX	Disc 1	4.71	2.64	128	128		0.26	7.181
		Disc 2	4.69	2.65	128				
B	MIN	Disc 1	4.51	2.59	120	121	2.34	0.26	7.148
		Disc 2	4.54	2.61	122				
	OPT	Disc 1	4.55	2.59	121	120.5		0.27	7.143
		Disc 2	4.52	2.58	120				
	MAX	Disc 1	4.52	2.53	117	116.5		0.26	7.319
		Disc 2	4.47	2.54	116				
C	MIN	Disc 1	4.47	2.54	116	116	4.32	0.27	7.117
		Disc 2	4.50	2.54	116				
	OPT	Disc 1	4.43	2.51	113	112.5		0.27	7.113
		Disc 2	4.39	2.50	112				
	MAX	Disc 1	4.30	2.44	107	106.5		0.26	7.111
		Disc 2	4.29	2.43	106				

**Table 5:** Results of the numerical simulations and real measurements (FRF) of the first natural frequency of the examined brake discs.

Cast Iron Grade	Chemical Composition	Disc Code	First Natural Frequency, Hz			Calculation Error, %
			Experiment		Simulation	
			Average Value	Standard Deviation		
A	OPT	Disc 1	1090	8.1	1098	0.73
		Disc 2	780	5.2	781	0.13
	MIN	Disc 1	1097	8.3	1106	0.82
		Disc 2	786	5.2	791	0.64
	MAX	Disc 1	1059	8.0	1066	0.66
		Disc 2	765	5.1	769	0.52
B	OPT	Disc 1	1033	8.0	1042	0.87
		Disc 2	742	5.1	748	0.27
	MIN	Disc 1	1054	8.0	1060	0.57
		Disc 2	751	5.0	753	0.27
	MAX	Disc 1	1034	7.8	1033	0.10
		Disc 2	733	5.0	735	0.81
C	OPT	Disc 1	1001	7.5	1016	1.50
		Disc 2	724	4.8	725	0.14
	MIN	Disc 1	1036	7.6	1031	0.48
		Disc 2	733	4.7	734	0.14
	MAX	Disc 1	980	7.5	994	1.43
		Disc 2	708	4.6	706	0.28
Average calculation error						0.63

**Table 6:** Results of the numerical modal analysis of the disc before and after blade modification.

Mode No	Natural Frequency, Hz		Difference, %
	Disc before Blade Modification	Disc after Blade Modification	
Mod I (0;2)	706	770	8.3
Mod II (0;3)	1676	1851	9.5
Mod III (0;4)	2751	3066	10.3

**Table 7:** Predicted and actual properties of the brake disc implemented in production.

Parameter	Predicted Value	Actual Value		Error, %
		Average Value	Standard Deviation	
Natural frequency, Hz	770	765	5.1	0.65
Young’s modulus, GPa	119	118.5	0.8	0.42
Tensile strength, MPa	222.4	218	7.7	2.01
Hardness, HB	202.9	204	6.1	0.53

## Data Availability

The original contributions presented in the study are included in the article, further inquiries can be directed to the corresponding author.

## References

[1] Pevec M., Potrc I., Bombek G. (2012). Prediction of cooling factors of a vehicle brake disc and its influence on results of thermal numerical simulation. Int. J. Automot. Technol..

[2] Vadiraj A., Balachandran G., Kamaraj M., Gopalakrishna B., Venkateshwara Rao D. (2010). Wear behavior of alloyed hypereutectic gray cast iron. Tribol. Int..

[3] Mackin T.J., Noe S.C., Ball K.J., Bedell B.C., Bim-Merle D.P., Bingaman M.C., Bingaman D.M., Bomleny G.J., Chemlir D.B., Clayton H.A. (2002). Thermal cracking in disc brakes. Eng. Fail. Anal..

[4] Pevec M., Oder G., Potrc I., Sraml M. (2014). Elevated temperature low cycle fatigue of grey cast iron used for automotive brake discs. Eng. Fail. Anal..

[5] Kchaou M., Sellami A., Elleuch R., Singh H. (2013). Friction characteristics of a brake friction material under different braking conditions. Mater. Des..

[6] Kumar N., Bharti A., Goyal H.S., Kant Patel K. (2021). The evolution of brake friction materials: A review. Mater. Phys. Mech..

[7] Borawski A. (2020). Conventional and unconventional materials used in the production of brake pads—Review. Sci. Eng. Compos. Mater..

[8] Szymański P., Czarnecka-Komorowska D., Gawdzińska K., Trubas A., Kostecka E. (2020). A review of composite materials used in brake disc pad manufacturing process. Compos. Theory Pract..

[9] Aranke O., Algenaid W., Awe S., Joshi S. (2019). Coatings for Automotive Gray Cast Iron Brake Discs: A Review. Coatings.

[10] Federici M., Menapace C., Moscatelli A., Gialanella S., Straffelini G. (2017). Pin-on-disc study of a friction material dry sliding against HVOF coated discs at room temperature and 300 degrees C. Tribol. Int..

[11] Menapace C., Mancini A., Federici M., Straffelini G., Gialanella S. (2019). Characterization of airborne wear debris produced by brake pads pressed against HVOF-coated discs. Friction.

[12] Krawczyk J. (2011). Microstructure and tribological properties of mottled cast iron with different chemical composition. Arch. Mat. Sci. Eng..

[13] Jakubus A., Soiński M.S. (2018). Thermal shock resistance of cast iron with various shapes of graphite precipitates. Arch. Foundry Eng..

[14] Casati R., Faccin R., Vedani M. (2018). Microstructural evolution and thermal fatigue resistance of grey cast iron. Fatigue Fract. Eng. Mat. Struct..

[15] Mróz M., Orłowicz A.W., Tupaj M., Burek M., Radoń M., Kawiński M. (2020). The Effect of Structure on Thermal Power of Cast-iron Heat Exchangers. Arch. Foundry Eng..

[16] Podrzucki C. (1991). Żeliwo. Struktura, Właściwości, Zastosowanie. (Cast Iron. Structure, Properties, Application).

[17] Elliott R. (1988). Cast Iron Technology.

[18] Rundman K.B. (2001). Cast Irons. Encyclopedia of Materials: Science and Technology.

[19] Masoud I.M., Al-Jarrah J.A., Abu Mansour T. (2014). Manufacturing of Gray Cast Iron Automotive Disc Brake. Indian J. Appl. Res..

[20] Djafri M., Bouchetara M., Busch C., Weber S. (2014). Effects of humidity and corrosion on the tribological behaviour of the brake disc materials. Wear.

[21] Xu W., Ferry M., Wang Y. (2005). Influence of alloying elements on as-cast microstructure and strength of gray iron. Mater. Sci. Eng. A.

[22] Tsujikawa M., Nagamine K., Ikenaga A., Hino M. (2008). Influence of graphite morphology on dry sliding wear of flake graphite cast irons. Int. J. Cast Met. Res..

[23] Polak A., Grzybek J. (2005). The mechanism of changes in the surface layer of grey cast iron automotive brake disc. Mater. Res..

[24] Maluf O., Angeloni M., Milan M.T., Spinelli D., Filho W.W.B. (2004). Development of materials for automotive disc brakes. Pesqui. Technol Minerva.

[25] Tonolini P., Montesano L., Pola A., Bontempi G., Gelfi M. (2023). Wear Behavior of Nb Alloyed Gray Cast Iron for Automotive Brake Disc Application. Metals.

[26] Konopka Z., Łągiewka M., Zyska A. (2020). Influence of Cast Iron Modification on Free Vibration Frequency of Casting. Arch. Foundry Eng..

[27] (2019). Microstructure of Cast Irons. Part 1: Graphite Classification by Bisual Analysis.

[28] Liles G.D. (1989). Analysis of Disc Brake Squeal Using Finite Element Methods.

[29] Liu P., Zheng H., Cai C., Wang Y.Y., Lu C., Ang K.H., Liu G.R. (2007). Analysis of disc brake squeal using the complex eigenvalue method. Appl Acoust..

[30] Kung S.W., Dunlap K.B., Ballinger R.S. (2000). Complex Eigenvalue Analysis for Reducing Low Frequency Brake Squeal.

[31] Belhocine A., Ghazaly N.M. (2016). Effects of Young’s Modulus on Disc Brake Squeal using Finite Element Analysis. Int. J. Acoust. Vib..

[32] Sinou J.J. (2010). Transient non-linear dynamic analysis of automotive disc brake squeal—On the need to consider both stability and non-linear analysis. Mech. Res. Commun..

[33] Nouby M., Sujatha C., Srinivasan K. (2011). Modelling of Automotive Disc Brake Squeal and Its Reduction Using Rotor Design Modification. Int. J. Veh Noise Vib..

[34] Genuit K. (2010). The Future of NVH Research—A Challenge by New Powertrains.

[35] (2014). Metallic Materials — Brinell Hardness Test Part 1: Test Method.

[36] (2010). Non-Destructive testing—Characterization and Verification of Ultrasonic Testing Equipment - Part 1: Instruments.

[37] (2010). Non-Destructive Testing - Characterization and Verification of Ultrasonic Examination Equipment - Part 2: Probes.

[38] (1975). Odlewnicze Stopy Aluminium i Miedzi. Badania Technologiczne. Pomiar Stopnia Zagazowania.

[39] (1975). Gray, Spheroidal Graphite and Malleable Cast Iron. Metallographic examination. Evaluation of microstructure.

[40] Wojciechowski A., Sobczak J. (2001). Kompozytowe Tarcze Hamulcowe Pojazdów Drogowych (Composite brake discs for road vehicles).

[41] Wehr J. (1972). Measurements of the Velocity and Attenuation of Ultrasonic Waves.

[42] (1996). Shewhart Control Charts.

